# Customizing workflows for electronic patient-reported outcome (ePRO) symptom monitoring using the action, actor, context, target, time (AACTT) framework

**DOI:** 10.1007/s11136-025-03995-y

**Published:** 2025-05-29

**Authors:** Julia Lai-Kwon, Claudia Rutherford, Michael Jefford, Iris Zhang, Catherine Devereux, Dishan Herath, Kate Burbury, Stephanie Best

**Affiliations:** 1 Department of Medical Oncology, Peter MacCallum Cancer Centre, Melbourne, Australia; 2 Department of Health Services Research, Peter MacCallum Cancer Centre, Melbourne, Australia; 3 Cancer Care Research Unit, Susan Wakil School of Nursing and Midwifery, Faculty of Medicine and Health, The University of Sydney, Sydney, Australia; 4 Sir Peter MacCallum Department of Oncology, University of Melbourne, Melbourne, Australia; 5 Australian Cancer Survivorship Centre, Peter MacCallum Cancer Centre, Melbourne, Australia; 6 Digital Healthcare Innovations Team, Peter MacCallum Cancer Centre, Melbourne, Australia; 7 School of Health Sciences, University of Melbourne, Melbourne, Australia; 8 Australian Genomics, Murdoch Children’s Research Institute, Melbourne, Australia

**Keywords:** Symptom monitoring, Patient-reported outcomes, Workflows, Implementation science, AACTT framework

## Abstract

**Background:**

Real-time electronic patient-reported outcome (ePRO) symptom monitoring is a complex intervention with few examples of successful implementation at scale. A key challenge is designing a clear ePRO symptom monitoring workflow to support implementation into practice. We aimed to create an empirical and theory-informed site-specific workflow guided by the Action, Actor, Context, Target, Time (AACTT) implementation science framework.

**Methods:**

A five-step process was undertaken to customize a generic ePRO symptom monitoring workflow to create a site-specific version: (1) design a generic ePRO symptom monitoring workflow through a qualitative study with key stakeholders; (2) conduct co-design workshops to understand stakeholder preferences regarding a site-specific version; (3) code co-design workshop data using the AACTT framework to produce a provisional site-specific version; (4) conduct a final co-design workshop using the AACTT framework to finalize stakeholder preferences for a site-specific version; and (5) code co-design workshop data using the AACTT framework to produce a final site-specific version.

**Results:**

Participants (n = 27) included nine patients, four caregivers, four oncologists, four nurses, two pharmacists, two clinic administrators, and two Electronic Medical Record (EMR) analysts. Provisional and final site-specific workflows were generated outlining the key AACTT components for each step of ePRO symptom monitoring.

**Conclusion:**

We demonstrated the value in using the AACTT to guide the co-design of a site-specific workflow for ePRO symptom monitoring. By describing this process in detail, we will enable others to replicate this process for creating site-specific workflows not only for ePRO symptom monitoring, but for any complex clinical process.

**Supplementary Information:**

The online version contains supplementary material available at 10.1007/s11136-025-03995-y.

## Background

Real-time electronic patient-reported outcome (ePRO) symptom monitoring is an evidence-based method of detecting cancer symptoms and treatment side-effects. Multiple studies have demonstrated improved symptom control, physical functioning, treatment adherence, patient satisfaction, patient self-efficacy, cost-effectiveness and overall survival, as well as reductions in emergency department presentations and hospital admissions [1, 2] when using ePRO symptom monitoring.

One of the key challenges in implementing ePRO symptom monitoring is designing a symptom monitoring workflow that aligns with existing work practices, staffing and resourcing [1, 3–5]. ePRO symptom monitoring often requires a variety of people to interact with the ePRO system to monitor patients remotely for symptoms and side-effects of their cancer and treatment. This process may involve patients, caregivers, nurses, doctors, administrative staff and information technology (IT) staff. Actions could include designated staff enrolling patients into ePRO symptom monitoring, patients and/or caregivers completing surveys regarding symptoms, automated alerts to clinical staff for severe or worsening symptoms, and staff contacting patients to discuss severe or worsening symptoms. Staff may also need to train patients and caregivers in using the ePRO system, provide technical assistance, and follow up missing surveys.

Whilst the aim is to integrate ePRO symptom monitoring using existing resources and processes, successful implementation also requires people to change some behaviors and work practices. To support this transition, it is helpful to describe each required task as clearly and consistently as possible to ensure everyone involved in symptom monitoring understands their specific role. Describing tasks clearly and consistently can also inform the development of patient and staff training, standard operating procedures, and protocols for ePRO symptom monitoring. In addition, process mapping of existing clinical workflows [6–9], followed by co-designing updated workflows incorporating ePRO symptom monitoring with stakeholders [10, 11] will ensure clinicians are able to efficiently and meaningfully integrate ePROs into their decision making.

Implementation science models, theories and frameworks have been identified as an important facilitator to guide implementation of ePRO symptom monitoring [7, 12–15]. However, there are few practical examples of how to use implementation science frameworks to prospectively design workflows for ePRO symptom monitoring.

Multiple implementation science theories and frameworks exist [16, 17], including process models that describe how to translate research into practice (e.g. Knowledge to Action [18, 19]), determinant frameworks that specify the barriers and facilitators that influence implementation outcomes (e.g. the Consolidated Framework for Implementation Research [20, 21]) and evaluation frameworks that evaluate the degree of implementation success (e.g. Reach Effectiveness Adoption Implementation and Maintenance (RE-AIM) framework [22, 23]).

In contrast, the Action, Actor, Context, Target, Time (AACTT) framework [24] is an implementation science framework used to specify behaviors within complex procedures. Each task is described in terms of the action needing to be performed, the actor who performs the action, the context in which the action occurs, the target for whom/ with whom the action is performed, and the time when the action is performed. It provides a structure for considering the key components of each step in a complex workflow. This information is critical for clear role delineation and task allocation to inform creation of site-specific protocols and support successful implementation. The AACTT framework therefore provides a vehicle to rigorously customize a generic workflow to create a site-specific version.

Use of the AACTT has been previously recommended in publications focusing on the implementation of ePRO symptom monitoring [12]. However, there are no published examples of how to do this. We aimed to create a site-specific workflow informed by the AACTT framework to demonstrate how it can be used to support implementation of ePRO symptom monitoring.

## Methods

This study was conducted at an Australian quaternary cancer center as part of a broader study to co-design an ePRO symptom monitoring system and its associated workflow for people receiving immune checkpoint inhibitors (ICI). Institutional ethics approval was obtained (HREC/96744/Peter MacCallum Cancer Centre). All participants provided written informed consent.

**Fig. 1:**
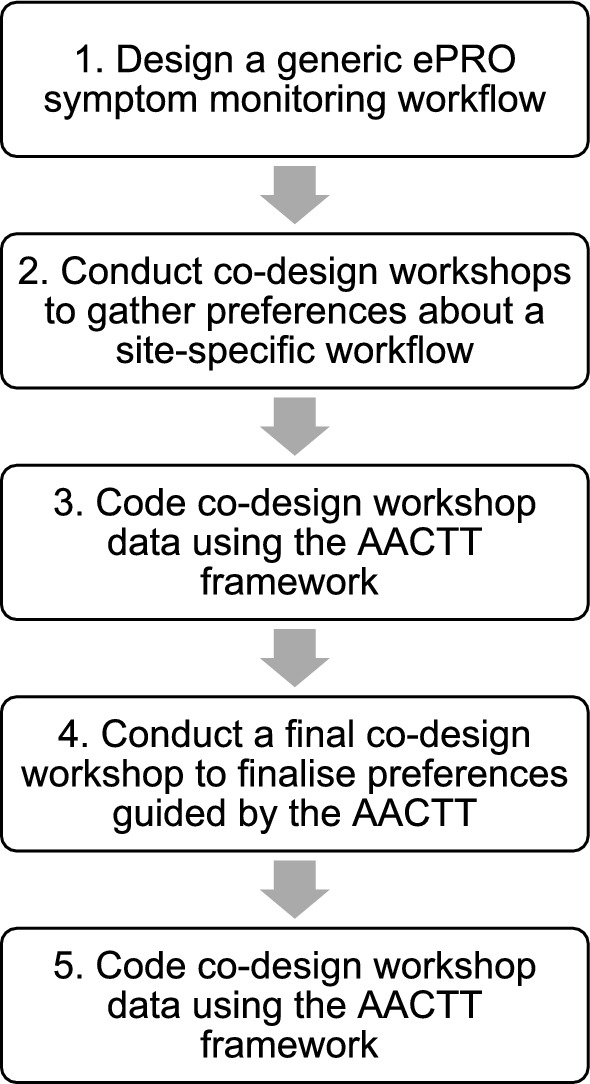
Five-step process for customizing a generic ePRO symptom monitoring workflow into a site-specific workflow

The following steps describe our process for customizing a generic ePRO symptom monitoring workflow into a site-specific version using the AACTT framework **(**Fig. 1**)**. The AACTT framework was used to code co-design workshop data to produce a provisional site-specific workflow (Step 3), inform the conduct of the third co-design workshop (Step 4), and coding of data (Step 5) from the third co-design workshop to produce the final site-specific workflow.

1. **Design a generic ePRO symptom monitoring workflow**
  The generic ePRO symptom monitoring workflow was designed during a preceding qualitative study [25]. In brief, this study aimed to identify barriers and facilitators to implementing ePRO symptom monitoring in routine cancer care using the Consolidated Framework for Implementation Research (CFIR) [20]. The study involved focus groups and individual interviews with 30 participants from the same Australian quaternary cancer center, including patients, caregivers, medical oncologists, nurses, hospital leaders, clinic administrators, pharmacists and IT specialists. Data was used to develop a generic workflow for how implementation strategies might be integrated into the planning, delivery and evaluation of ePRO symptom monitoring (Fig. 2). Participants were not asked about site-specific preferences during this study. Therefore, the workflow required further customization to develop a site-specific workflow.

**Fig. 2:**
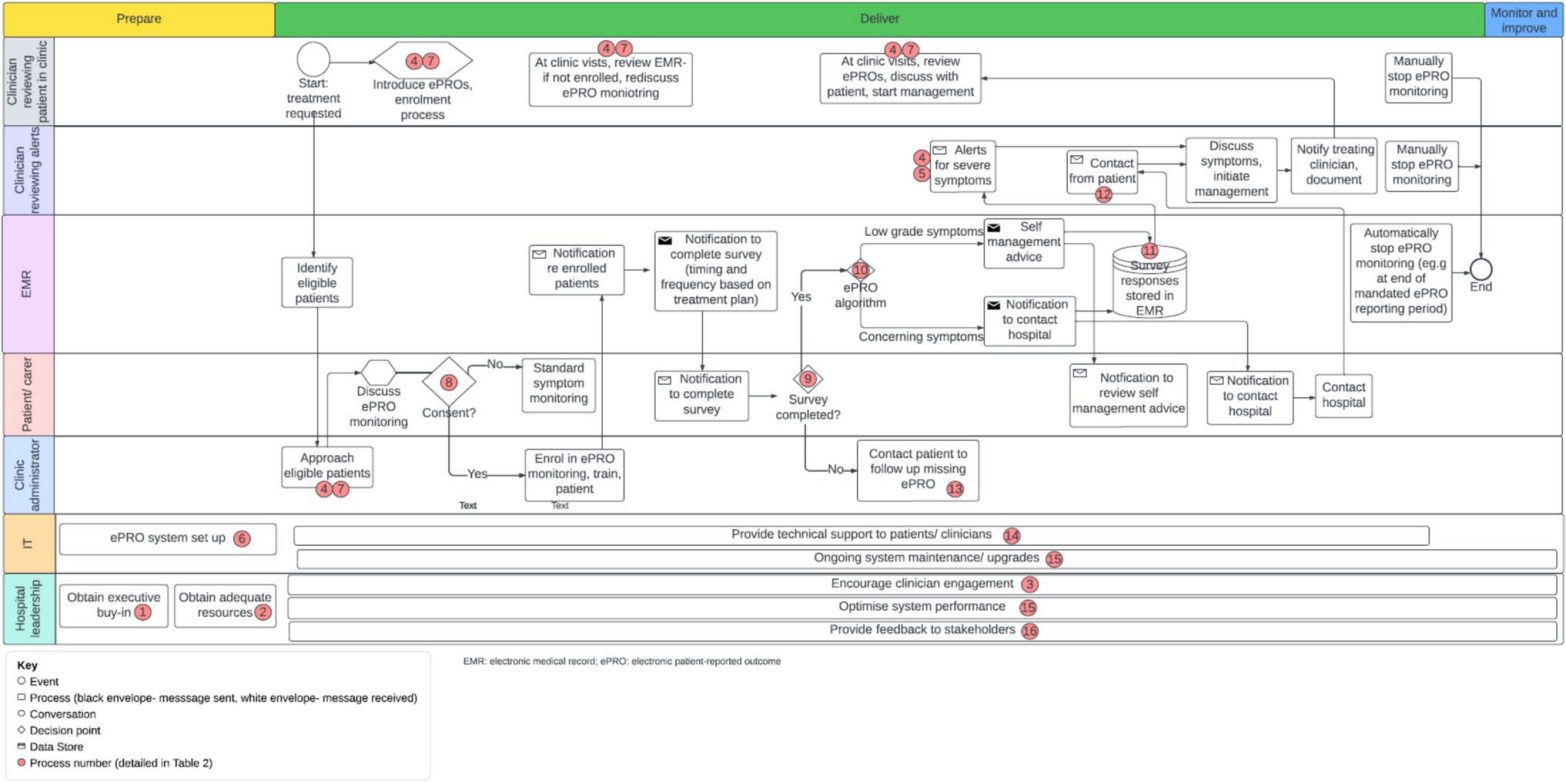
Generic ePRO symptom monitoring workflow
2. **Conduct co-design workshops to gather preferences about a site-specific workflow**
  We conducted two co-design workshops at an Australian quaternary cancer center to gather ideas regarding a site-specific ePRO symptom monitoring workflow.

- **Study population**
  Patient and caregiver participants were ≥ 18 years old and receiving or had previously received ICI (or had direct caring responsibility for a person who was receiving or who had previously received ICI). Clinician participants were oncologists, nurse specialists, pharmacists or administrative support staff involved in caring for people receiving ICI. EMR analyst participants were EMR analysts involved in the development and maintenance of the cancer center’s EMR (Epic, Verona, Wisconsin, USA). All participants attended at least one co-design workshop and were encouraged to attend the entire series.
  Participants were identified via a previously conducted qualitative study on barriers and facilitators to ePRO symptom monitoring [25] and contacted directly to discuss this study. Additional patient/caregiver participants were identified via melanoma and lung clinic lists, given that many patients in these clinics had experience of ICIs. These people were initially approached by their treating physician who obtained verbal consent for them to be contacted by a research assistant. The study research assistant (IZ) then called the patient/ caregiver to discuss the study and arrange consent. Additional clinicians and EMR analysts were identified via the researchers’ professional networks and emailed the information and consent form. To ensure a diverse range of viewpoints and to account for drop out, we aimed to recruit 10–15 patients and caregivers and 10–15 clinicians/EMR analysts.
- **Co-design workshops 1 and 2**
  We used co-design workshops to capture iterative feedback to inform the provisional site-specific workflow (Fig. 2). Workshop 1 focused on ideation around the ‘ideal’ site-specific ePRO symptom monitoring workflow. Workshop 2 asked participants to discuss further ideas and preferences around a site-specific workflow. We did not present the previously designed generic workflow or formally introduce the AACTT framework to participants during workshops 1 or 2 because we wanted to allow them to spontaneously generate ideas without being limited to the generic workflow or the AACTT framework. Each workshop was held three times (i.e. 6 workshops were conducted in total) to ensure that all participants were able to attend. Participants were encouraged, but not expected, to attend both workshops.
- **Data collection**
  The co-design workshops were co-facilitated by JLK (a medical oncologist), CD (an experienced co-design facilitator), and IZ (a research assistant). Prior to each workshop, all participants were provided with reading materials outlining the aims of the session, questions to be addressed, and anticipated outputs. Each workshop lasted 1.5 h and was conducted face-to-face or via Zoom or Microsoft Teams depending on participant preferences. Each workshop was audio and video recorded and transcribed using the automated transcription feature on Zoom/ Microsoft Teams. Transcripts were checked against audio/video recordings to ensure their accuracy and de-identified. Participants were not invited to review their transcripts but consented to being contacted after the workshops if further clarification of their comments was required. Participants were reimbursed for their attendance in accordance with the hospital’s consumer engagement policy.
  3. **Code co-design workshop data using the AACTT framework**
    A deductive approach was used to analyze transcripts from workshops 1 and 2 according to the steps within the generic ePRO symptom monitoring workflow outlined in step 1 and the AACTT framework described by Presseau et al. [24]. As each workshop was conducted three times, the first two (67%) transcripts for each workshop were read line by line by JLK and IZ to familiarize themselves with the dataset. JLK and IZ then coded these transcripts independently to the components of the AACTT framework (action, actor, context, target, time) using a deductive approach. Any discrepancies were resolved by discussion with co-authors. The remainder of the transcripts were then coded by IZ, reviewed by JLK, and any discrepancies were resolved by discussion with co-authors.
    Coding was undertaken in NVivo (QSR international, version 12). Data was displayed in a matrix where each row denoted a step in the generic ePRO symptom monitoring workflow, and each column denoted a component of the AACTT framework. All researchers were involved in data interpretation and final interpretations were arrived at by consensus. All findings were substantiated by the most representative quotes.
    This process resulted in a provisional site-specific workflow, with each step of the workflow described in terms of the AACTT. If participants described multiple options for the action, actor, context, target or time, these were retained.
  4. **Conduct a final co-design workshop to finalize preferences**
    The aim of co-design workshop 3 was to customize the generic ePRO symptom monitoring workflow into a site-specific version using the AACTT framework. Participants were first shown a Microsoft PowerPoint presentation with the provisional site-specific workflow with each step described in terms of the AACTT. Participants were then divided into mixed stakeholder groups consisting of patients, caregivers, clinicians and EMR analysts of up to 6 people with 1 facilitator (JLK, IZ, or CD). Each group was asked to review up to three steps of the provisional site-specific workflow, discuss and select their preferred action, actor, context, target and time for the local context. JLK and IZ allocated steps to review to each group to ensure that each step was considered by at least two groups to ensure a range of perspectives were captured. Data collection was performed as per step 2.
  5. **Code co-design workshop data using the AACTT framework**
    A deductive approach was again used to analyze transcripts from workshop 3 according to the steps within the generic ePRO symptom monitoring workflow and the AACTT framework [24]. The process for analyzing the transcripts was as per step 3. This resulted in a final site-specific version, with each step described in terms of preferred action, actor, context, target and time for the local context.

## Results

### Co-design workshop participant characteristics

Twenty-seven participants were recruited: nine patients (P), four caregivers (C), four oncologists (O), four specialist nurses (N), two pharmacists (Ph), two clinic administrators (A), and two EMR analysts (E). Participant characteristics are shown in Table 1: 17 (63%) were female; most were aged 61–70 years old (9, 33%); with English spoken at home (24, 89%); representing a range of cancer types.

**Table 1:** Co-design participant characteristics

		n	%
Sex	Male	10	37%
	Female	17	63%
Age group	18–30	1	
	31–40	6	
	41–50	5	
	51–60	3	
	61–70	9	
	> 70	3	
Country of birth	Australia	21	78%
	Other	6	22%
Language spoken at home	English only	24	89%
	Other	3	11%
Role	Patient	9	
	Caregiver	4	
	Oncologist	4	
	Nurse	4	
	Pharmacist	2	
	Clinic administrator	2	
	EMR analyst	2	
Cancer type ^#^*	Melanoma	12	
	Lung	1	
	Other	3	
Tumor stream (clinicians only)*	Melanoma	6	
	Lung	2	
	Gynecological	2	
	Prostate	2	
	Bladder	2	
	Kidney	2	
	Breast	1	
	Cancer of Unknown Primary	1	
	Role is not cancer type specific	4	
Duration of immune checkpoint inhibitor treatment^#^	< 6 months	1	
	6–12 months	5	
	13–18 months	4	
	> 18 months	3	
Duration of experience in current role (clinicians only)	1–4 years	8	
	5–9 years	2	
	10–14 years	2	
	> 15 years	2	
Access to Health Hub^#^	Yes	13	100%
Easy to ask doctors questions regarding their health^#^*	Strongly agree	13	93%
	Slightly agree	1	7%
Easy to ask nurses questions regarding their health ^#^*	Strongly agree	12	86%
	Slightly agree	2	14%
Confidence in working with doctors to manage immunotherapy side-effects ^#^	Very confident	8	
	Quite confident	4	
	Somewhat confident	1	
Use of electronic devices^#^*	Smartphone	13	
	Tablet	9	
	Desktop or laptop	9	
Self-reported confidence in using electronic devices for online activities ^#^*	Not at all confident	1	
	A little confident	3	
	Somewhat confident	4	
	Quite confident	3	
	Very confident	3	

EMR: electronic medical record ^#^Patients and caregivers only; * some participants provided multiple answers

### Co-design workshops 1 and 2

Co-design workshops 1 and 2 were each conducted three times from October 2023 to December 2023. Six transcripts were generated. Three workshops were conducted face-to-face and three were online. The median duration of each workshop was 84.5 min (range: 76–92 min).

Supplementary Table 1 shows data from co-design workshops 1 and 2 coded according to the steps of the generic ePRO symptom monitoring workflow (rows) and the AACTT (columns).

### Provisional site-specific workflow

**Fig. 3:**
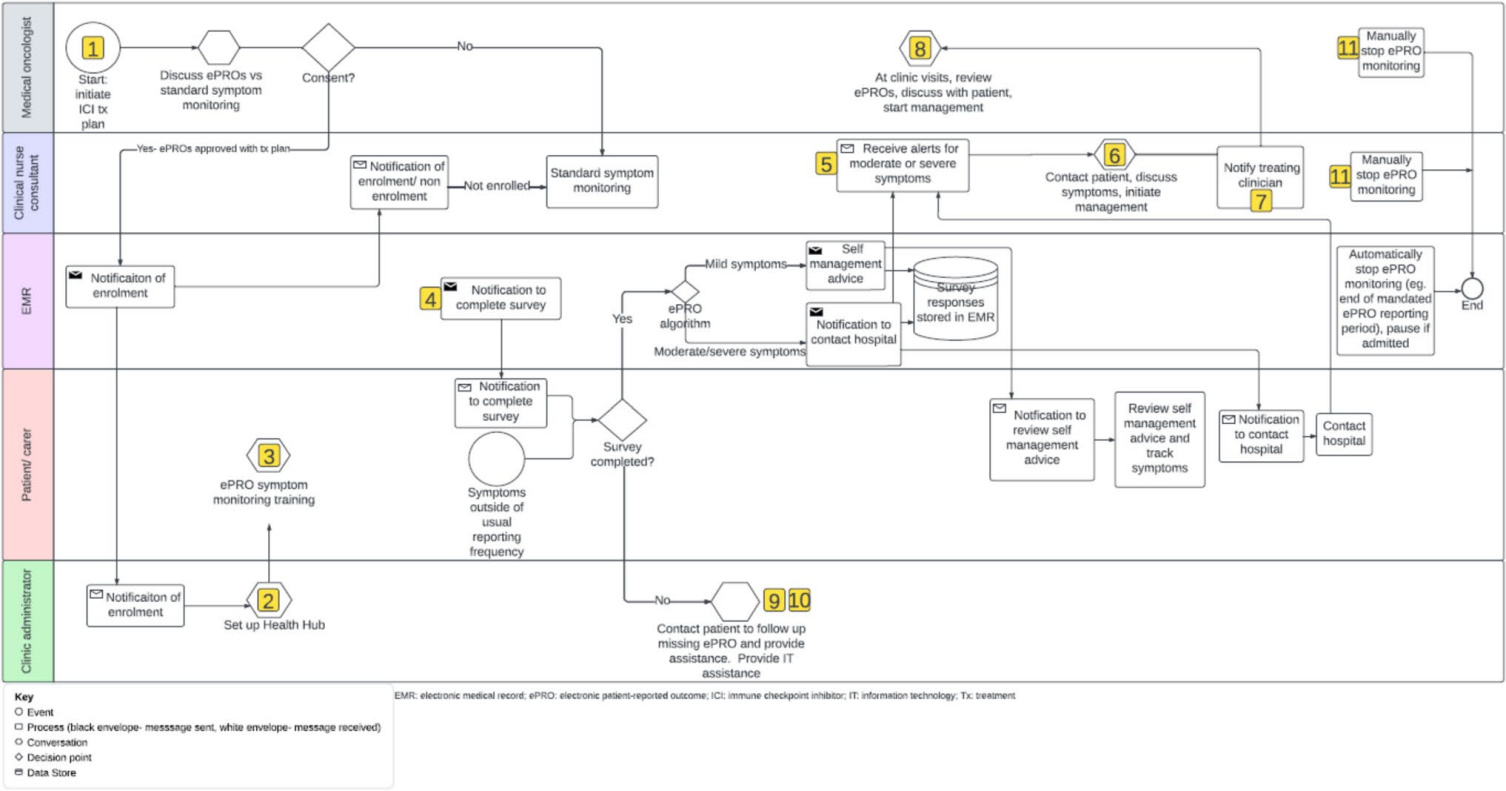
Provisional site-specific ePRO symptom monitoring workflow

Figure 3 shows the provisional site-specific workflow based on the findings from co-design workshops 1 and 2, with each step described in terms of the AACTT (Table 2). For each of the components of the AACTT, all options described by participants are displayed. Key differences between Figs. 2 and 3 include greater role specification (e.g. ‘clinician reviewing patient in clinic’ is clarified as the ‘medical oncologist’ in Fig. 3), additional steps (e.g. setting up of the Health Hub app and ePRO symptom monitoring training), changes to enrolment procedures (re-allocated to the medical oncologist rather than the clinic administrator) and removal of certain roles (e.g. IT and hospital leadership, as Fig. 2 included preparation and monitoring and improvement of ePRO symptom monitoring, rather than focusing specifically on delivery).

**Table 2:** Provisional site-specific ePRO symptom monitoring workflow steps described according to the AACTT

	Action Specify the behavior that needs to change in terms that can be observed or measured	Actor Specify the person/ people that do(es) or could do the action targeted	Context Specify the physical location, emotional context, or social setting in which the action is performed	Target Specify the person/ people with/for whom the action is performed	Time Specify when the action is performed (the time, date, frequency)
1. Initiate ICI treatment plan	Commence ICI-specific treatment plan within Epic Beacon module	Medical oncologist initiating treatment plan	In clinic room (via phone, telehealth or F2F)	Patient starting ICI	During medical oncology appointment when C1 of treatment plan is signed
2. Set up Health Hub	Patients can self-enroll in Health Hub via existing enrolment procedure (via a generic link or in person at hospital) If patient cannot self-enroll, enroll onto Health Hub on preferred device, ensure can log into Health Hub, demonstrate how to use Health Hub	*Options:* • Patient • Clinic administrator • Wellbeing center volunteer	*Options:* • At home • At hospital- clinic waiting room, clinic room, wellbeing center	Patient ± caregiver/ proxy requiring access to Health Hub	Prior to C1D1
3. ePRO symptom monitoring training	Instruct patient in how to report symptoms using ePRO system. Complete baseline survey, interpret immediate actions, view graphs, view self-management advice; what to do if additional concerns; who to call for IT help. Provide written information	*Options:* • Patient • Nurse • Clinic administrator • IT specialist	*Options:* • At home • At hospital- Day Therapy Unit in treatment chair, Day Therapy Unit in separate room	Patient ± caregiver/ proxy who will assist with Health Hub	*Options:* • Any time between initiating treatment plan and C1D1 • At treatment education visit in Day Therapy Unit
4. Notification to complete survey	Send out surveys via Health Hub	Epic (via Care Companion)	At home (via email, SMS, or push notification according to patient’s communication preferences in Health Hub)	Patient ± caregiver/ proxy	As soon as step 1 completed. First survey to be completed at baseline during step 3, then weekly for the first 3 months + ad hoc, then ad hoc
5. Receive alerts for moderate and severe symptoms	Receive alerts for moderate and severe symptoms	*Options:* During business hours: • Nurse • Doctor • Clinic administrator After hours: • Nurse • Doctor • Clinic administrator	*Options:* • At hospital (via email, text message, Secure Chat in Epic, in Basket message in Epic, or Epic dashboard)	Patient ± caregiver/ proxy who completed survey	*Options:* • During business hours • 24 h a day • In real-time as surveys are completed • At specific times of day
6. Contact patient, discuss symptoms, initiate management	Call patient ± caregiver/ proxy to assess symptoms and document discussion in Epic	*Options:* During business hours: • Nurse • Doctor • Clinic administrator After hours: • Nurse • Doctor • Clinic administrator	*Options*: • At hospital (via phone, telehealth, or F2F)	Patient ± caregiver/proxy who completed survey	*Options:* • In real-time as alerts are received • At specific times of day
7. Notify treating clinician	Notify treating clinician regarding moderate and severe alerts	*Options:* • Epic (automated process) • Managing nurse, doctor or clinic administrator	*Options:* • At hospital (via email, text message, Secure Chat in Epic, In Basket message in Epic or)	Treating clinician (patient’s primary medical oncologist)	Once a moderate or severe alert has been managed
8. At clinic visits, review ePROs, discuss with patient, start management	Review ePRO results since last clinic visit. Show patient graphs, discuss management of side-effects. If not completed, find out why and provide assistance	Medical oncologist	In clinic room (via phone, telehealth, or F2F)	Patient ± caregiver/ proxy	During pre-treatment review
9. Contact patient to follow up missing ePRO	Call patients who have missed their weekly ePRO. Offer assistance	*Options:* • Clinic administrator • Nurse	*Options:* • At hospital (via phone, telehealth, or F2F)	Patients ± caregiver/ proxy who have missed a weekly ePRO by ≥ 1 week	*Options:* • Daily during business hours • Weekly during business hours
10. Provide IT assistance	Provide technical assistance	*Options:* • IT specialist • Clinic administrator • Wellbeing center volunteer	*Options:* • At hospital (via phone, telehealth or F2F)	Patients ± caregiver/ proxy	*Options:* • During business hours • 24 h a day
11. Manually stop ePRO monitoring	Manually stop ePRO monitoring within Epic. This could occur due to cessation of treatment (for progression or toxicity), patient/ clinician preference	*Options:* • Nurse • Doctor	*Options* • At hospital (via phone, telehealth, or F2F)	Epic	*Options:* • At clinic visits • Between clinic visits

C1: cycle 1; C1D1: cycle 1 day 1; ePRO: electronic patient-reported outcomes; F2F: face to face; ICI: immune checkpoint inhibitor; IT: information technology

### Co-design workshop 3

Co-design workshop 3 was conducted three times from January-March 2024, resulting in three transcripts. Two workshops were conducted face to face and one was online. The median duration of each workshop was 90 min (range: 87 to 94 min).

Supplementary Table 2 shows data from co-design workshop 3 coded according to the steps of the provisional ePRO symptom monitoring workflow (rows) and the AACTT (columns).

### Final site-specific workflow

**Fig. 4:**
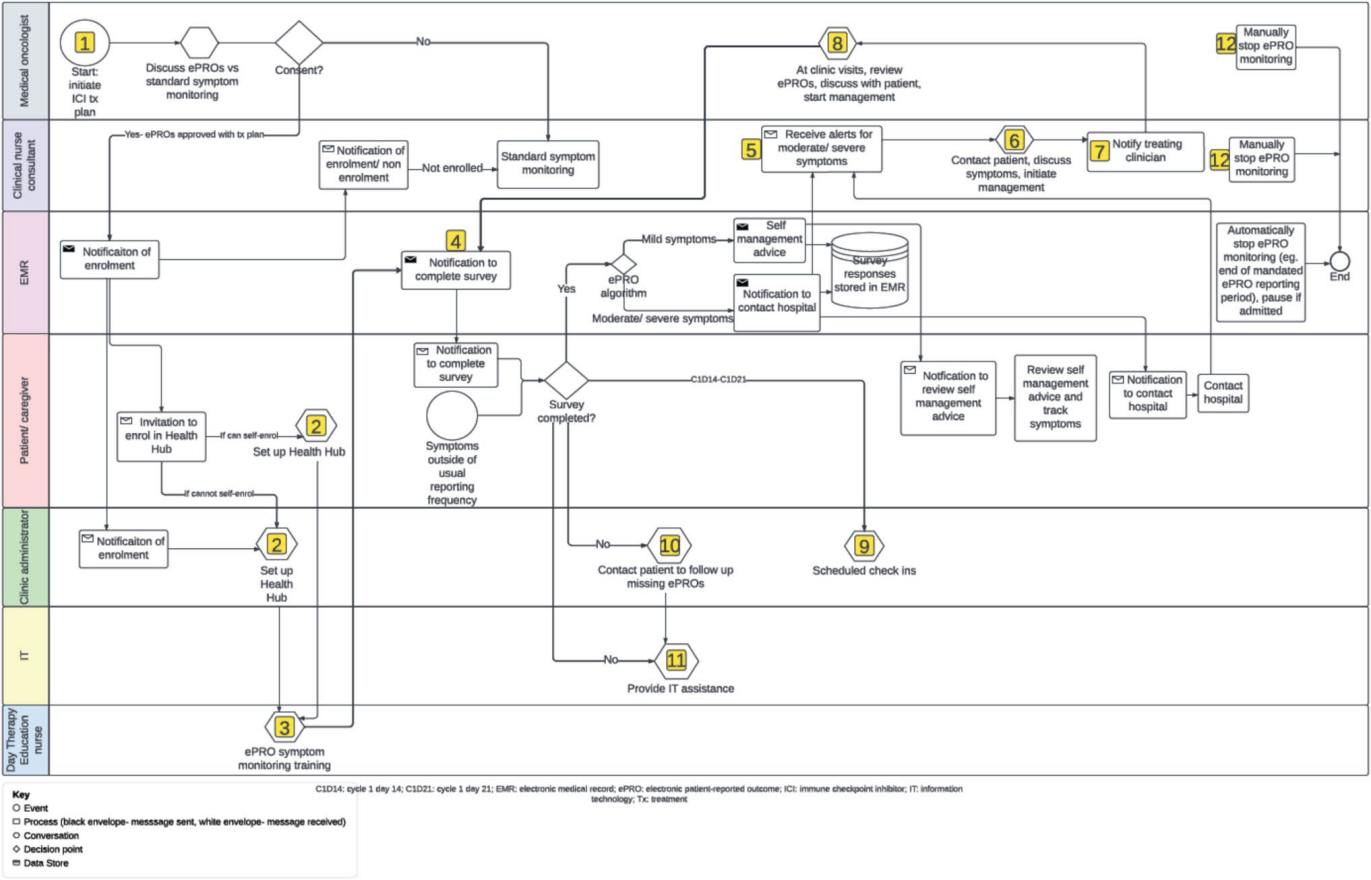
Final site-specific ePRO symptom monitoring workflow

Figure 4 shows the final site-specific workflow with each step described in terms of the AACTT (Table 3). For each of the components of the AACTT, all options described by participants are displayed. Some key differences between Figs. 3 and 4 include re-allocation of the role of ‘ePRO symptom monitoring training’ from the patient/caregiver to a Day Therapy Education Nurse to provide better support to patients/ caregivers and better fit with existing workflows, enabling patients/ caregivers and clinic administrators to set up Health Hub (the patient-facing app associated with the Epic electronic medical record) depending on ability to self-enroll, the addition of scheduled check ins by the clinic administrator to ensure patients are completing ePROs, and the re-addition of IT to provide IT assistance.

**Table 3:** Final site-specific ePRO symptom monitoring workflow steps described according to the AACTT

	Action Specify the behavior that needs to change in terms that can be observed or measured	Actor Specify the person/ people that do(es) or could do the action targeted	Context Specify the physical location, emotional context, or social setting in which the action is performed	Target Specify the person/ people with/for whom the action is performed	Time Specify when the action is performed (the time, date, frequency)
1. Initiate ICI treatment plan	Commence ICI-specific treatment plan within Epic Beacon module	Medical oncologist initiating treatment plan	In clinic room (via phone, telehealth or F2F)	Patient starting ICI	During medical oncology appointment when C1 of treatment plan is signed
2. Set up Health Hub	Patients can self-enroll in Health Hub via existing enrolment procedure (via a generic link or in person at hospital) If patient cannot self-enroll, enroll onto Health Hub on preferred device, ensure can log into Health Hub, demonstrate how to use Health Hub	If patient can self-enroll: patient ± caregiver/ proxy If patient cannot self-enroll: clinic administrator OR Wellbeing Center volunteer	If patient can self-enroll: at home If patient cannot self-enroll: at hospital (prefer F2F in clinic waiting room or Wellbeing Center, otherwise phone)	Patient ± caregiver/ proxy requiring access to Health Hub	Between step 1 and C1D1
3. ePRO symptom monitoring training	Instruct patient in how to report symptoms using ePRO system. Complete baseline survey, interpreting immediate actions, view graphs, view self-management advice; what to do if additional concerns; who to call for IT help. Provide written information	Day Therapy Unit nurse	At hospital- Day Therapy Unit in treatment chair or separate room (depending on availability)	Patient ± caregiver/ proxy who will assist with Health Hub	At treatment education visit
4. Notification to complete survey	Send out surveys via Health Hub	Epic (via Care Companion)	At home (via email, SMS, or push notification according to patient’s communication preferences in Health Hub)	Patient ± caregiver/ proxy	As soon as step 1 completed. First survey to be completed at baseline during step 3, then weekly for the first 3 months + ad hoc, then ad hoc
5. Receive alerts for moderate and severe symptoms	**Moderate alerts:** • *Patient action*: call clinical nurse consultant within 24 h during business hours • *Clinical nurse consultant action*: o *Business hours:* non-urgent alert on Epic dashboard. Review twice/day at set times (e.g. 10am/ 4 pm, determined by clinical nurse consultant’s existing work practices) during business hours o *After hours:* non-urgent alert on Epic dashboard. Review during business hours **Severe alerts:** • *Patient action*: call hospital immediately. If emergency, call 000 o *Business hours:* call clinical nurse consultant o *After hours:* call after hours coordinator • *Clinician action*: o *Business hours*: alert to clinical nurse consultant via Epic Haiku notification to duty phone. Call patient back immediately o *After hours*: alert to after-hours coordinator via Epic Haiku notification to duty phone. Call patient back immediately				
6. Contact patient, discuss symptoms, initiate management	Call patient ± caregiver/ proxy to assess symptoms and document discussion in Epic	*Business hours*: clinical nurse consultant *After hours*: after hours coordinator	At hospital (via phone, telehealth, or F2F)	Patient ± caregiver/proxy who completed survey	Time frames as per step 5
7. Notify treating clinician	Notify treating clinician regarding moderate and severe alerts	*Business hours*: clinical nurse consultant *After hours*: after hours coordinator	At hospital (via email)	Primary medical oncologist OR medical oncologist who last reviewed the patient	Once a moderate or severe alert has been managed
8. At clinic visits, review ePROs, discuss with patient, start management	Review ePRO results since last clinic visit. Show patient graphs, discuss management of side-effects. If not completed, find out why and provide assistance	Medical oncologist	In clinic (via phone, telehealth, or F2F)	Patient ± caregiver/ proxy	During pre-treatment review
9. Scheduled check ins	Check in with patient ± caregiver/ proxy to reinforce ePRO and troubleshoot any issues	Clinic administrator	In hospital (via phone or F2F)	Patient ± caregiver/ proxy	Once after complete 1st survey independently (C1D14 to C1D21)
10. Contact patient to follow up missing ePROs	Call patients who have missed their weekly ePRO. Assist with manual completion if required	Clinic administrator	At hospital (via phone or F2F)	Patients ± caregiver/ proxy who have missed a weekly ePRO by ≥ 1 week	Review ePRO dashboard once a week during business hours
11. Provide IT assistance	Provide technical assistance. Requests for assistance can come from the patient, caregiver/ proxy or clinicians	Wellbeing Center volunteer or IT specialist	At hospital (via phone or F2F in the Wellbeing Center)	Patients ± caregiver/ proxy	During business hours as required
12. Manually stop ePRO monitoring	Manually stop ePRO monitoring within Epic. This could occur due to cessation of treatment (for progression or toxicity), patient/ clinician preference	Medical oncologist or clinical nurse consultant	At hospital (via phone, telehealth, or F2F)	Epic	Anytime during ePRO symptom monitoring

C1: cycle 1; C1D1: cycle 1 day 1; ePRO: electronic patient-reported outcomes; F2F: face to face; ICI: immune checkpoint inhibitor; IT: information technology

## Discussion

Implementing ePRO symptom monitoring is a complex process. While designing a site-specific ePRO symptom monitoring workflow is only one part of this process, it remains one of the key challenges of implementing ePRO symptom monitoring. Multiple guidelines on implementing ePRO symptom monitoring highlight the importance of mapping current workflows and processes, followed by careful re-design of existing workflows to accommodate ePRO symptom monitoring [8, 12, 26, 27]. However, there are no published examples of how to practically do this.

This study aims to address this gap by providing a practical example of how to co-design a site-specific workflow with key stakeholders using an implementation science framework (the AACTT framework). Building on a generic ePRO monitoring workflow, we used the AACTT framework to guide the customization of this workflow according to local needs and preferences. This approach ensured the final site-specific workflow aligned with existing workflows and utilized existing staff. Describing this process in detail will help support the development of standard operating procedures and patient and clinician training to facilitate real-world implementation of ePRO symptom monitoring. It will also enable others tasked to produce site-specific workflows for ePRO symptom monitoring to replicate the optimization of generic guidance.

Utilizing the AACTT framework enabled us to thoroughly consider details about the steps required to perform ePRO symptom monitoring that had not been previously identified. For example, in co-design workshops 1 and 2 where participants spontaneously described their ‘ideal’ ePRO symptom monitoring workflow, many participants focused on the actor who should be performing the action as well as the action itself. However, the other components of the AACTT (context, target, time) required to establish a service were often not spontaneously described by participants as reflected by the gaps within Supplementary Table 1. Using the AACTT framework explicitly in workshop 3 enabled participants to consider the context, target and time for each step. Capturing critical service delivery information highlights the need for using a specific framework, such as the AACTT, to provide structure and ensure completeness of the customization process.

The AACTT framework facilitated the identification of differences in perspectives between key stakeholders and for these to be openly discussed as a group. For example, in step 3 of the provisional site-specific workflow (‘ePRO symptom monitoring training’), different participants suggested different ‘actors’ who should be responsible for carrying out this step including patients, nurses, clinic administrator and IT specialists. Using the AACTT to uncover differences in opinion regarding the ideal ‘actor’ for this step helped guide the discussion during workshop 3 and ensure that a single preferred actor was selected by the group. This process provided role clarity and how it could be carried out in practice. However, in some instances such as step 12 of the final site-specific workflow (‘manually stop ePRO monitoring’), two options for the ‘actor’ (a medical oncologist or clinical nurse consultant) were both considered feasible in daily clinical practice and were selected, allowing for flexibility with this step.

The AACTT helped participants conceptualize the details required for the future implementation of ePRO symptom monitoring in clinical practice. For example, during discussions in co-design workshops 1 and 2 regarding the ideal ‘actor’ to perform steps 5 and 6 (‘receive alerts for moderate and severe symptoms’, ‘contact patient, discuss symptoms and initiate management’), several possibilities were noted, but some debate arose as to who might be the best person at different times of the day. In co-design workshop 3, the use of the AACTT framework, with its emphasis on actor as well as time and context, helped participants to realize that the ‘actor’ could be different depending on whether the symptom was noted during or outside of usual business hours (time, context). This helped participants to identify a specific actor, time and context for during versus outside of usual business hours. This information allowed the alignment of the final site-specific workflow with current staffing and workflows during, versus outside of, usual business hours, and ensured there were clear expectations about how this critical step was managed at different times of the day.

This work has several strengths. Firstly, this is one of the first published descriptions of how the AACTT can be used to develop site-specific workflows for complex clinical processes (prior examples include COVID-19 vaccine delivery [28] and maternal healthcare delivery [29]). We have provided a practical example of how to use the AACTT framework to break down complex processes into clear steps with measurable outcomes. By specifying the core elements of each task, the AACTT makes implicit thoughts and expectations explicit, ensuring everyone understands ‘who’, ‘what’, ‘where’, ‘when’ and ‘how’ things need to be done. In turn, this can help identify and address barriers to change, and evaluate whether strategies to address these barriers have worked [24]. Using the AACTT to specify behaviors can improve their interpretability and thus people’s adherence. This can be applied not just to ePRO symptom monitoring, but to any complex intervention comprised of multiple steps that require a degree of behavior change. Finally, the work highlights the importance of diverse stakeholder input into the customization of workflows. All stakeholders brought different understandings of the ideal action, actor, context, target and time to the initial discussions in co-design workshops 1 and 2, which informed the subsequent discussion in co-design workshop 3. However, the AACTT also ensured that all stakeholders ultimately reached consensus on a preferred action, actor, context, target and time to move this into reality.

Limitations of this work include the multiple steps within our proposed methodology for customizing a generic workflow into a site-specific workflow (co-design workshops, creation of provisional and final workflows) which may not always be feasible to perform, particularly in busy clinical environments. The time and costs associated with stakeholder participation in the co-design process was not collected as part of this study. Time-based activity costing could be included as part of future studies to ensure the type and amount of upfront investment required is described. However, the rigorous use of the AACTT does demonstrate its value in guiding this process by ensuring all components of every step are systematically considered, which may help support future implementation. Furthermore, the AACTT may help to narrow down areas where information is lacking, thus helping to focus effort and target discussions. Understanding how to perform this process rigorously may assist clinical teams to undertake an abbreviated process for co-designing their site-specific workflows, whilst still focusing on the critical components identified by the AACTT. Less resource intensive approaches to co-design are available, such as one-off focus groups with or without document review [30]. However, this would only be appropriate if minor local context revisions were required for the workflow. Secondly, we did not specifically measure the degree of inter-rater reliability for each component of the AACTT at each stage of the process. Finally, this workflow is yet to be implemented in routine care and its feasibility and acceptability are yet to be assessed. This will be conducted as part of future work.

For those seeking to use the AACTT framework for customization of generic workflows into site-specific workflows in the future, we would emphasize the importance of using the framework as early as possible within the co-design process. Early inclusion of the AACTT will ensure participants are orientated to focusing on the components of the AACTT from the beginning to maximize idea generation around each AACTT component. Where possible, multiple workshops should be run with as many participants as is feasible to ensure all perspectives are reflected in the final workflow. We would suggest prioritizing a small number of steps within your workflow for discussion, rather than trying to discuss all steps during each workshop, to maximize time for participant discussion and ensure all AACTT components are comprehensively discussed.

## Conclusion

We demonstrated that the AACTT implementation science framework can be used to co-design site-specific workflows for complex processes such as ePRO symptom monitoring. By outlining our process for using the AACTT framework, this can be replicated by not just those interested in creating site-specific workflows for ePRO symptom monitoring but is also generalizable to developing site-specific workflows for other complex clinical processes or interventions. We highlight the strengths of using the AACTT to specify the components within each step, thus supporting future real-world implementation, and highlight practical lessons learned from using the AACTT.

## Supplementary Information

Below is the link to the electronic supplementary material.

Supplementary file1 (XLSX 19 kb)

Supplementary file2 (XLSX 22 kb)

## Data Availability

De-identified participant data underlying the results reported in this manuscript will be shared. Data will be available immediately following publication with no end date. Investigators who propose use of the data that has been approved by an independent review committee will be granted access to the data to achieve the aims of the approved proposal. Proposals should be directed to julia.lai-kwon@petermac.org.
